# Ultrasound-Responsive Nanocarriers for Breast Cancer Chemotherapy

**DOI:** 10.3390/mi13091508

**Published:** 2022-09-11

**Authors:** Gelan Ayana, Jaemyung Ryu, Se-woon Choe

**Affiliations:** 1 Department of Medical IT Convergence Engineering, Kumoh National Institute of Technology, Gumi 39253, Korea; 2 Department of Optical Engineering, Kumoh National Institute of Technology, Gumi 39253, Korea; 3 Department of IT Convergence Engineering, Kumoh National Institute of Technology, Gumi 39253, Korea

**Keywords:** ultrasound, nanocarriers, micro-/nano-bubbles, breast cancer, chemotherapy

## Abstract

Breast cancer is the most common type of cancer and it is treated with surgical intervention, radiotherapy, chemotherapy, or a combination of these regimens. Despite chemotherapy’s ample use, it has limitations such as bioavailability, adverse side effects, high-dose requirements, low therapeutic indices, multiple drug resistance development, and non-specific targeting. Drug delivery vehicles or carriers, of which nanocarriers are prominent, have been introduced to overcome chemotherapy limitations. Nanocarriers have been preferentially used in breast cancer chemotherapy because of their role in protecting therapeutic agents from degradation, enabling efficient drug concentration in target cells or tissues, overcoming drug resistance, and their relatively small size. However, nanocarriers are affected by physiological barriers, bioavailability of transported drugs, and other factors. To resolve these issues, the use of external stimuli has been introduced, such as ultrasound, infrared light, thermal stimulation, microwaves, and X-rays. Recently, ultrasound-responsive nanocarriers have become popular because they are cost-effective, non-invasive, specific, tissue-penetrating, and deliver high drug concentrations to their target. In this paper, we review recent developments in ultrasound-guided nanocarriers for breast cancer chemotherapy, discuss the relevant challenges, and provide insights into future directions.

## 1. Introduction

Breast cancer surpassed lung cancer as the most common cancer (in terms of new incidence of cancer). In 2020 alone, there were 2.26 million new cases and 685,000 deaths from breast cancer worldwide [1]. Early diagnosis and better treatment have been shown to reduce the mortality rate by 1%, each year from 2013 to 2018 [2]. There are three main treatment methods for breast cancer: surgical intervention, whereby the tumor tissue is removed before it metastasizes to other locations; radiation therapy, whereby high-energy waves are utilized to destroy cancer cells; and chemotherapy, whereby therapeutic agents are used to destroy cancer cells or shrink tumors [3]. These treatment methods can sometimes be used in combination [3].

Chemotherapy (chemo), a cancer treatment method that uses anti-cancer drugs (chemotherapeutic agents), is the most widely used systemic breast cancer treatment for suppressing cancer cell proliferation and for its ability to move all around the body and destroy a wide range of cancers [4]. However, chemotherapy has limitations. Most chemotherapeutic agents exert their effects by halting mitosis (cell division), targeting the fast dividing (proliferating) cells, and causing damage to cells (cytotoxic effect) [5]. The cytotoxic effects of chemotherapeutic agents are not only limited to cancer cells. They also destroy normal cells, leading to adverse side effects such as immunosuppression, myelosuppression, neutropenic enterocolitis, gastrointestinal distress, anemia, nausea and vomiting, hair loss, secondary neoplasm, infertility, teratogenicity, peripheral neuropathy, cognitive impairment, tumor lysis syndrome, and organ damage among others [6]. Moreover, the bioavailability of therapeutic agents to tumor tissues is low, which warrants a higher number of drugs than needed, resulting in increased toxicity in normal cells and a high rate of multiple drug resistance [7]. Therefore, targeted drug delivery (TDD) enables effective delivery of therapeutic agents that increase the presence of drugs in a particular part of the body relative to others. TDD compensates for the limitations of conventional chemotherapy [7,8] and has been achieved using a drug carrier.

Drug carriers or vehicles are substrates used in chemotherapy to improve the specificity, bioavailability, and safety of anti-cancer drugs [9]. Drug carriers are important for regulating the release of therapeutic agents into the body [10]. They can act through diffusion, in which a slow release of a therapeutic agent occurs over a long period of time, or through a triggered release at the agent’s target with the help of external stimuli, such as changes in pH, heat, and light [10]. Nanoparticle (NP) drug carriers or nanocarriers have many advantages and, thus, have been widely used as drug carriers for breast cancer chemotherapy. Their efficient pharmacokinetics, precise targeting of tumor cells, and reduced side effects make nanoparticles the best therapeutic agent carriers in breast cancer chemotherapy [6]. Furthermore, nanoparticles have merits, such as passive targeting and distant circulation [11]. Unfortunately, nanoparticles are also affected by physiological barriers and partially by the bioavailability of therapeutic agents [12]. Moreover, nanoparticles have limitations regarding lack of biodegradation and potential toxicity in the case of long-term administration [13].

**Figure 1:**
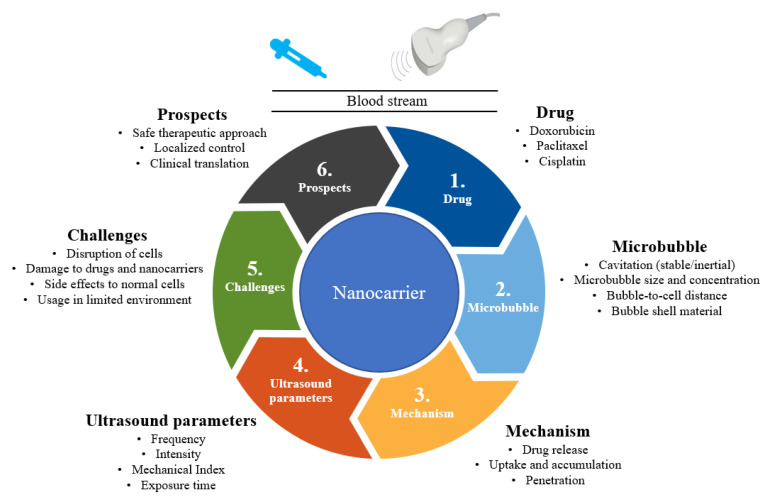
Summary of ultrasound-responsive nanocarriers for breast cancer chemotherapy.

To address these challenges, approaches involving external stimulation with light, ultrasound, heat, microwaves, and X-rays have been explored [14,15,16]. In particular, studies on ultrasound-based targeted drug delivery for breast cancer chemotherapy have attracted considerable attention in the past few years. This could be due to ultrasound’s properties such as cost-effectiveness, non-invasiveness, specificity, tissue penetration, and achieving high drug concentrations at their target [17,18,19]. This review discusses recent developments in ultrasound-stimulated nanocarriers for breast cancer chemotherapy. Furthermore, the current challenges and future directions are sought. There are a few reviews on the issue of ultrasound-based targeted drug delivery for cancer treatment in general. In contrast to that, we focused on ultrasound-guided nanoparticle drug carriers for breast cancer chemotherapy. We summarize the overall concept of ultrasound-responsive nanocarrier-based breast cancer therapy in Figure 1.

## 2. Nanocarriers for Breast Cancer Chemotherapy

**Figure 2:**
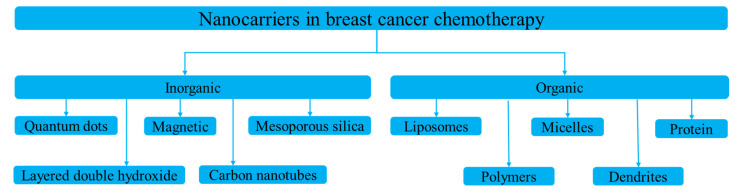
Types of nanocarriers used in breast cancer chemotherapy.

Nanoparticles designed for either targeted or non-targeted drug delivery have a small diameter (1–100 nm) and possess a large surface area to volume ratio [20]. These properties allow them to bind, absorb, and carry therapeutic agents with high efficiency [21]. Nanocarriers for breast cancer chemotherapy are broadly divided into two types: organic and inorganic (Figure 2) [22]. Inorganic nanocarriers include quantum dots (QD), mesoporous silica nanoparticles (MSN), layered double hydroxide (LDH) nanoparticles, carbon nanotubes, and magnetic nanoparticles. Inorganic nanocarriers are preferred for their better anti-cancer agent-loading capacity, large surface area, reduced side effects, bioavailability, well-regulated drug release, and—most importantly—for their organic solvent tolerance. Organic nanocarriers, on the other hand, include polymeric nanoparticles, liposomes, micelles, protein nanoparticles, and dendrites. Organic nanocarriers are preferred for their easy synthesis and modification, enabling improved drug-loading efficacy, biodistribution, and therapeutic efficacy. Moreover, organic nanoparticles allow sustained drug release over a period of time and the use of organic solvents [9].

The most commonly used nanocarriers in breast cancer chemotherapy include liposomes, dendrimers, micelles, carbon nanotubes, polymeric nanoparticles, solid lipid nanoparticles (SLNs), and nanostructured lipid carriers (NLCs) [12]. Liposomes are used for various purposes to increase drug-loading capacity while suppressing unnecessary drug effects. In contrast, lipids cause toxicity, and nanocarriers are quickly destroyed by phagocytes. Dendrimers have been commended for their higher loading capacity and bioavailability. However, dendrimers suffer from rapid clearance, organ accumulation, and synthesis variability. Micelles reduce toxicity and other side effects, but are used only for limited drugs and exhibit low drug-loading capacity [11]. Carbon nanotubes are capable of penetrating and localizing at the cellular level, but as a material, they can be potentially toxic. Polymeric nanoparticles are biocompatible, degradable, non-toxic; however, they are less effective and susceptible to carrier degradation. SLNs have the advantage of being soluble and better controlled for drug release, despite their low drug-loading capacity and containing other complex structures [23]. NLCs have multiple advantages compared to others, and their limitations include gelation of lipid dispersion and polymorphic transition. In general, nanocarriers for breast chemotherapy have their advantages and shortcomings. To improve their shortcomings while increasing treatment efficacy, different stimuli are utilized, which are designed to make the nanocarriers responsive.

## 3. Stimuli-Responsive Nanocarriers for Breast Cancer Chemotherapy

**Figure 3:**
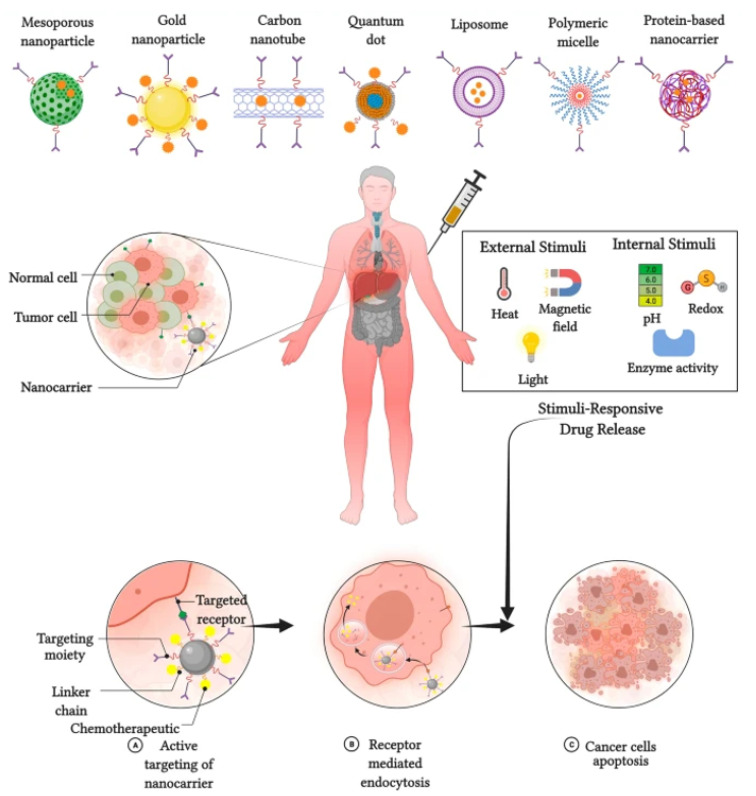
Stimulus responsive nanocarriers. Adapted from Kaushik et al. [10] with permission under the terms of the CC BY 4.0 License, Copyright 2022.

The application of stimuli to improve the efficacy of therapeutic agents delivered by nanocarriers has received considerable attention in recent years. Stimuli-responsive nanocarriers have been developed to compensate for the shortcomings of conventional nanocarrier-based chemotherapy [8]. The delivery of therapeutic agents responsive to stimuli is based on both internal (endogenous) and external (exogenous) stimuli (Figure 3).

### 3.1. Internal Stimuli

Various internal stimuli are used for nanocarrier-based anti-cancer agent delivery to increase therapeutic efficacy and suppress adverse effects. Internal stimuli used with nanocarriers in breast cancer chemotherapy include pH, redox, and enzymatic stimuli [15]. pH-responsive nanocarriers are internalized and dissociate, causing protonation and extracellular drug release. Subsequently, the nanoparticle is detached, which promotes endocytosis of nanocarriers and release of the drug [24]. The redox-responsive nanocarrier system is the S–S bond that is chemically cross-linked as a gating or capping molecule on the surface of the nanoparticle and is cleaved upon the addition of agents, causing rapid drug release to the tumor cells [25]. Drug release from NPs in an enzyme-responsive manner originates from specific enzyme-catalyzed chemical reactions that lead to the degradation, dissociation, or morphological transitions of the parent NPs [14].

### 3.2. External Stimuli

External stimuli originate from outside of the body to initiate anti-cancer agent delivery. External stimuli used in breast cancer chemotherapy include magnetic fields, ultrasound, and light [26]. In contrast to the internal stimuli, the external stimuli would introduce contrast agents to the image—that of nanoparticles located in the target tissues, cells, or organelles. This further triggers nanocarriers from outside the body through particular stimuli at a specific time. Magnetic systems are widely utilized for targeting and imaging [27]. As magnetic-responsive nanotherapeutics are non-invasive signals, an externally applied magnetic field can damage the moving particles and increase the accumulation of therapeutic agents in tumors. A magnetic field could be employed for in vivo applications, and could have greater advantages for targeted cancer therapy as compared with intrinsic stimuli-responsive nanotherapeutics. Ultrasound is one of the most commonly used exogenous stimuli in cancer therapy [28]. The unique advantages of ultrasound responsiveness include safety, non-invasiveness, and deeper penetration into the tissue. Many exogenous stimuli are used for drug delivery systems, among which temperature-responsive drug delivery systems offer potential advantages compared to other counterparts. This is due to their flexible design, regulation of phase transition temperatures, and passive targeting capability. The localized hyperthermia from 42.5 to 43.5 °C helps to evade cancer cells by inducing high temperatures in tumor tissues. However, these hyperthermic stimuli would enlarge the blood vessels and modify the perforation of tumor cell membranes, thereby enhancing anti-tumor drug delivery [29].

### 3.3. Internal vs. External Stimuli

Both internal and external stimuli have their own advantages and disadvantages, as presented in Table 1. Internal stimuli are safe and provide efficient and controllable drug release without compromising cell and site specificity. Internal stimuli have the disadvantage of not being controlled manually. External stimuli have the advantage of being manually controlled and modulated based on individual requirement making it vital in personalized treatment. They also provide upgraded site-specific drug delivery and enable regulated and payload release. However, external stimuli need more sophisticated equipment and normal cell injury may happen. Nevertheless, compared to internal stimuli, external stimuli are preferred for nanocarrier based chemotherapy.

## 4. Biological Effects of Ultrasound

The effect of ultrasound waves on tissues or cells is significant for breast cancer treatment [30]. There are two broad categories of ultrasound effects on tissues or cells in therapeutic applications. The first is the thermal effect, whereby a continuous application of ultrasound on a target tissue or cell increases the temperature of the tissue or cell. Ultrasound can be applied to create a low- or high-temperature depending on the therapeutic application. The second is mechanical, whereby high-energy ultrasound affects cells or tissues mechanically; for example, causing them to vibrate. The ultrasound-induced biological effects (either by thermal or mechanical means) can be categorized into four primary mechanisms: thermal, cavitation, acoustic streaming, and bilayer sonophore effects.

### 4.1. Thermal Effect

The thermal effect of ultrasound is primarily an increase in the temperature of the medium owing to the absorption of energy from ultrasound waves. The rate of heat generated by ultrasound waves is directly proportional to the frequency of the waves and exposure time and is inversely proportional to the specific absorption coefficient of the targeted tissue. Consequently, the higher the absorption coefficient of the medium, the more significant the increase in temperature and—in turn—the thermal effect experienced by the tissue. The thermal dose delivered to a cell or tissue quantifies the magnitude and duration of the temperature change. Ultrasound energy can result in local hypothermia (creating either a low-level thermal rise over several minutes or hours) [3] or, conversely, can result in thermal ablation (a short duration highly localized high temperature rise that destroys the tissue via protein denaturation).

### 4.2. Cavitation Effect

The application of ultrasound waves in the tissue environment causes a pressure change, resulting in the formation of bubbles in a phenomenon called cavitation [31,32]. The cavitation effect induced by ultrasound includes the formation, growth, oscillation, and collapse of cavities in the tissue environment stream owing to pressure changes following the induced ultrasound wave [33]. Cavitation can occur in both endogenous and exogenous gas bubbles. Endogenous gas microbubbles are naturally occurring cavities in the cell cytoplasm, whereas exogenous gas vesicles comprise synthetic gas vesicles or microbubbles introduced into the cellular microenvironment from the outside [34]. They consist of spherical cavities filled with gas and/or saturated carbon and are typically stabilized by an encapsulated surfactant, phospholipids, and a synthetic polymer or denatured human serum albumin. The inner and outer microbubbles can increase the permeability of cell membranes through pore formation on the membrane. This leads to acoustophoresis and is considered to be a cavitation induced by sonoporation [35]. Microbubbles can cause sonoporation when the cavitation thresholds are reached. When ultrasound is applied to microbubbles, it absorbs ultrasonic energy and causes high-frequency oscillations. As a result, a liquid jet or shock wave is formed, leading to disruption of the cell membrane structures. The oscillation and expansion of microbubbles exert shear pressure on the cell membrane, which also increases the permeability of the cell membrane; hence, this process makes cells more accessible to nanoparticles [36].

**Figure 4:**
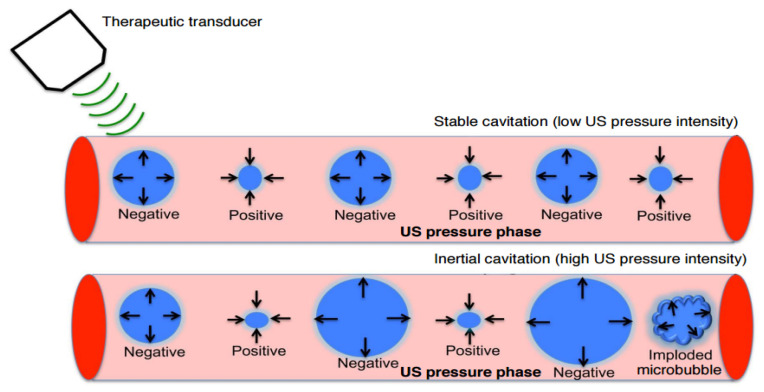
Stable and inertial cavitation. Adapted from Chowdhury et al. [32] with permission under the terms of the CC BY-NC 3.0 License, Copyright 2017.

The two types of cavitation that exist, as shown in Figure 4, depend on how bubbles collapse when subjected to ultrasound: stable and inertial cavitation [37,38]. Stable cavitation is a non-linear, perennial, periodic bubble expansion and contraction. In stable cavitation, the gas pockets present in the liquid oscillate around an equilibrium radius and can persist for a long period of time. This can cause gas microbubbles to shrink and expand under the influence of ultrasound for a long period. The microbubble oscillation period lasts until the gas content of the microbubble dissolves in the blood, and then it is rapidly cleared through exhalation from the lungs. Inertial cavitation, on the other hand, is characterized by a violent collapse of bubbles whereby the collapse of bubbles occurs instantly with the applied ultrasound wave [39]. Inertial cavitation induces larger pore sizes than stable cavitation does.

### 4.3. Acoustic Streaming Effect

When ultrasound waves with high amplitudes are applied to a medium, the transfer of momentum from the ultrasound wave to the medium may lead to the generation of unidirectional flow of currents in the fluid, a phenomenon known as acoustic streaming [40]. The velocity of the stream is directly proportional to the attenuation coefficient of the medium, ultrasound intensity, and surface area of the transducer and is inversely proportional to the speed of sound in the medium in question and the bulk viscosity. The leading cause of acoustic streaming is the ultrasound reflection and other distortions that occur during wave propagation [41]. To date, the clinical value of acoustic streaming has only been minimally explored. In general, acoustic streaming is produced by a non-linear acoustic wave with a finite amplitude propagating in a fluid. The molecules are forced to oscillate at the same frequency as the acoustic waves to which they are exposed. As a result of the non-linearity of the acoustic wave, wave propagation results in a time-independent flow velocity on top of the regular oscillatory motion. Therefore, the fluid moves in a particular direction that depends on the structure of the system and its boundary conditions as well as the parameters of the applied ultrasound wave. The smaller acoustic streams in a fluid is referred to as microstreaming [42]. When associated with acoustic cavitation, which refers to the activities of microbubbles in a general sense, it is referred to as cavitation microstreaming [43].

### 4.4. Bilayer Sonophore Effect

In ultrasound-responsive therapy, nanocarriers containing bubbles filled with gas are injected into the bloodstream. The interaction of ultrasound waves with the bubbles result in a temporary increase in the permeability of the bilayer membranes. Once the ultrasound wave reaches the target region of interest, it activates the bubble within the bloodstream at the target location. The ultrasound waves interact with the bubble, which absorbs acoustic energy, causing it to expand and contract within the capillaries. This phenomenon is called the bilayer sonophore effect, which results in the stretching and compression of the capillary walls [44]. The protein complexes are mechanically separated, and the tight junctions on the membranes become unlocked. As the permeability of the bilayer membrane increases, the therapeutic agent begins to exit the capillaries and enter the tumor tissue. The duration of the sonophore effect is very short, lasting only one or two minutes. The barrier remains permeable for only a few hours after the sonophore effect stops [45], widening intracellular spaces and facilitating paracellular transport. Transcellular transport can also increase as endothelial cells shuttle drugs out of the vessel lumen. This drug is now able to reach its therapeutic target. As the procedure resolves, bubbles disappear from the circulation within minutes. In 24 h or less after the sonophore effect, the junction proteins re-associate, the endothelial cells are restored to their original state, and the barrier fully regains its protective function. Through this effect, a variety of drugs can gain access to the target tumor for treatment, while minimizing the area and duration of the enhanced tumor-blood permeability.

## 5. Mechanism of Ultrasound-Responsive Nanocarriers in Breast Cancer Chemotherapy

### 5.1. Ultrasound-Responsive Nanocarrier Structure

**Figure 5:**
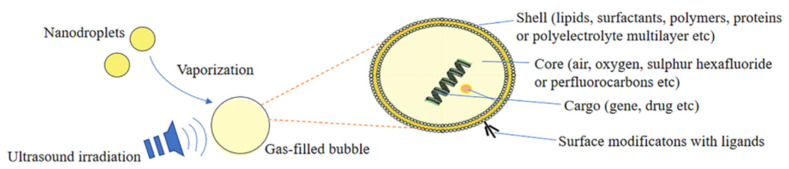
Structure of ultrasound-responsive nanocarrier. Adapted from Lu et al. [36] with permission under the terms of the CC BY 4.0 License, Copyright 2022.

Ultrasound-responsive nanocarriers function via a phenomenon called micro-/nano-bubbles, which are filled with gas. The bubble structure used in the ultrasound-responsive nanocarrier mechanism is composed of four main parts: the shell, core, cargo, and surface modification ligands (see Figure 5) [36].

For drug delivery applications, therapeutic drug-carrying microbubbles can be fabricated by incorporating the drug into or on bubbles. There are various ways in which drugs can be incorporated into acoustic carriers, ranging from association with the membrane to the development of gas- or drug-filled microspheres. The gas in drug delivery vehicles helps to create acoustic activity, which lowers the threshold for cavitation, making the drug carriers more sensitive to ultrasound for local activation, drug release, and drug delivery.

### 5.2. Ultrasound-Responsive Stimuli Nanocarriers Based Therapeutic Agent Delivery Phenomenon

**Figure 6:**
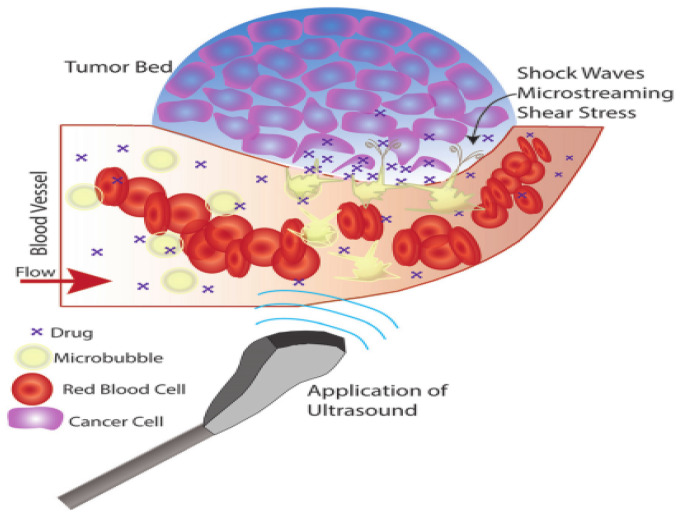
Ultrasound-responsive nanocarrier mechanism. Adapted from Delaney et al. [46] with permission under the terms of the CC BY 3.0 License, Copyright 2022.

Ultrasound-responsive nanocarrier-based therapeutic agent delivery is mostly achieved by a phenomenon called microbubbles, which consist of a gas core covered by a stabilizing shell of nanocarriers, as shown in Figure 6 [46]. Microbubbles facilitate the targeting of therapeutic agents through their unique interaction with ultrasound stimuli [47]. With the application of ultrasound stimuli, microbubbles injected intravenously expand and contract, leading to a phenomenon called cavitation, which occurs due to the acoustic impedance difference between the blood and the gas core [28].

Depending on the applied ultrasound stimuli, microbubbles behave in three ways during breast cancer chemotherapy [48]. First, for a low mechanical index (MI) of less than 0.2, the ultrasound stimuli cause bubbles to vibrate, resulting in small ruptures in cell membranes at the target, enhancing therapeutic agent delivery locally. Second, for a moderate increase in MI between 0.2 and 0.8, larger ruptures in capillaries result in the escape of blood, pooling, and increase in drug uptake caused by changes in vasculature permeability occurs in a phenomenon called sonoporation. Third, a further increase in MI greater than 0.8 leads to a disruption of microbubbles, resulting in the excitation of shock waves from microbubbles in a phenomenon called cavitation.

### 5.3. Therapeutic Agent Delivery Mechanism via Ultrasound-Responsive Nanocarriers

The nanocarriers’ primary job is to deliver therapeutic agents to the desired location. A variety of mechanisms—including passive diffusion, particle phagocytosis, pinocytosis, and receptor-mediated endocytosis—are frequently used in the transport of drugs to cells [49]. This is achieved through either active targeting or passive targeting [50,51]. In passive targeting, the transport of the drug-loaded nanocarriers into the tumors is facilitated by passively targeting, which takes advantage of the increased endothelial blood microvasculature permeability in tumors caused by the bigger interstitial gaps between the neighboring cells [52]. Due to limited removal in the tumor, this increased penetration and retention (EPR) effect permits larger drug accumulation as well as longer drug exposure duration [29]. To target particular cancer cells, active targeting systems use the EPR effect and targeting ligands that are covalently bound to the surface of nanocarriers [21,52,53]. These targeting ligands are particular to a certain cell surface biomarker or receptor molecules that are overexpressed in malignant cells [54]. Vitamin receptors, αvβ3 integrin receptor, PSMA (prostate-specific membrane antigen) receptor, growth factor receptor, insulin and insulin-like receptors, choosing protein molecules, and transferrin are among of the typical surface indicators that are overexpressed in malignancies and inflammatory illnesses. Folic acid (FA, vitamin B9) receptors (FAR-, FAR-), riboflavin (vitamin B2) receptors, and biotin receptors go under the category of vitamins, whereas fibroblast growth factor receptors (FGFR) and epidermal growth factor receptors (EGFR) are under the category of growth factors. Since the process for endocytic uptake of these targeted carriers needs the combined occurrences of several contemporaneous contacts at the contact point of numerous pairs of surface receptor and ligand, the targeting ligand is often linked to the nanocarrier surface in multiple copies. The tight adhesion between the nanocarriers and the targeted cell surface is attained during the receptor-mediated absorption of the nanocarriers by the targeted cell as a result of the multivalent binding mechanism.

**Figure 7:**
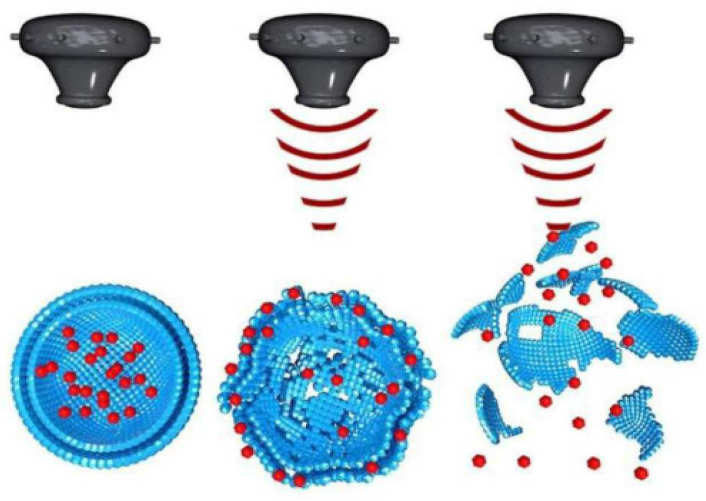
Ultrasound stimulated disruption of nanocarriers. Adapted from Tharkar et al. [55] with permission under the terms of the CC BY 4.0 License, Copyright 2019.

There are three mechanisms by which ultrasound is utilized to enhance the delivery of therapeutic agents for breast cancer chemotherapy via nanocarriers: triggering drug release from nanocarriers, uptake and accumulation, and penetration of nanocarriers [55]. In the case of triggering drug release, ultrasound disrupts nanocarriers (Figure 7) via two effects: thermal and non-thermal effects [56]. In this mechanism, nanocarriers are made to respond to the mechanical effects of ultrasound waves, the thermal effect of ultrasound waves, or both effects together. Thus, ultrasound facilitates the local release of drugs from nanocarriers at the tumor site. The use of ultrasound helps minimize the dosage and suppress side effects on healthy cells. In the case of the uptake and accumulation mechanism, upon ultrasound stimuli, the microbubbles are injected into the bloodstream and induce mechanical forces against the blood vessel wall, leading to the diffusion of nanocarriers and drugs towards the tumor extracellular matrix. This resolves the problem of low and proportional uptake of nanoparticles in the tumor tissue. Finally, in the case of nanocarrier penetration, ultrasound stimuli solve the problem of drug penetration in solid tumors by pushing the nanoparticles into the tumor area, thereby improving accumulation and deeper penetration. This resolves the current limitations of chemotherapy, such as low drug concentration, toxicity to healthy cells, and other side effects.

## 6. Ultrasound-Responsive Nanocarriers for Breast Cancer Treatment

A few studies have applied ultrasound-responsive nanocarriers in breast cancer chemotherapy to improve treatment. Table 2 summarizes some studies related to ultrasound-responsive nanocarriers for breast cancer treatment. Wu et al. [29] developed Pluronic P123/F127 polymeric micelles encapsulating curcumin, which were stimulated by focused ultrasound to trigger drug release. They achieved longer circulation and increased uptake with the application of ultrasound. Jablonowski et al. [57] combined surface tumor necrosis factor (TNF)-related apoptosis-inducing ligand (TRAIL) expression and doxorubicin co-encapsulation in the form of ultrasound-responsive microbubbles to improve the treatment effects in breast cancer cell lines. They reported that ultrasound resulted in the greatest reduction in cancer cell survival, while shielding the destruction of healthy MCF-12A cells. Eisenbrey et al. [58] utilized localized microbubbles to suppress hypoxia prior to breast cancer therapy. In their study, surfactant-shelled oxygen microbubbles were fabricated and injected intravenously to locally elevate tumor oxygen levels when triggered by non-invasive ultrasound in mice with human breast cancer tumors. Furthermore, Soyemi et al. [59], Wang et al. [60], Dobruch-Sobczak et al. [61], Rix et al. [62], and Yang et al. [56] demonstrated the application of ultrasound along with contrast agents in breast cancer chemotherapy to improve patients undergoing neoadjuvant chemotherapy. These studies found that an ultrasound-based approach enabled the accurate identification of responding and non-responding tumors. Amioka et al. [63] proposed ultrasound-responsive chemotherapy for neoadjuvant breast cancer, and concluded that ultrasound-responsive methods might serve as a new approach for planning therapeutic strategies for patients with breast cancer after neoadjuvant chemotherapy. Baghbani et al. [37] loaded alginate-stabilized nanodroplets with doxorubicin for ultrasonic theranostics. They reported that their proposed approach possessed highly enhanced anti-cancer effects under ultrasound and displayed long-lasting, strong ultrasound contrast. Bush et al. [64] also studied the therapeutic efficacy of ultrasound-responsive nanocarriers with liposomal doxorubicin. They observed improved treatment efficacy with acoustic cluster therapy. Delaney et al. [65] investigated whether ultrasound-induced rupture of microbubbles improves breast cancer metastasis and reported that adding ultrasound-ruptured microbubbles to radiation therapy delays tumor progression and enhances the survival rates of patients with metastatic breast cancer. Nie et al. [66] employed a multimodal approach for targeted breast cancer imaging, in which a tumor was subjected to a strong echo signal via ultrasound. They were able to see an improved performance when using the multimodal approach. Sheng et al. [67] proposed ultrasound-guided breast therapy using nanodroplets and reported that nanodroplets with tunable properties enable efficient site-specific drug delivery and exhibit their potency in cancer theranostics. Song et al. [68] proposed ultrasound-responsive delivery to control cell proliferation. They reported a positive effect of using ultrasound and microbubbles for breast cancer, and that their approach could be translated to other types of cancer treatment.

## 7. Therapeutic Agents in Ultrasound-Responsive Breast Cancer Treatment

Therapeutic agents are chemical substances that are delivered to the body for the treatment or mitigation of disease conditions or ailments. These substances can be drugs, proteins, genes, compounds, or other pharmaceutically active ingredients. As the human genome has been sequenced and genetic technology has advanced, there is a growing body of knowledge on genetic changes, initiation and proliferation, therapeutic mechanisms, and novel treatment targets for cancer therapy. Understanding the pathophysiology of the disease, human gene sequences, and discovery of novel molecular targets is the core of modern medicine to conquer cancer therapy. Numerous noteworthy advances have been made in the development of targeted therapy. These targeted therapies are designed to attack cancer cells while causing less damage to normal healthy cells. Targeted therapies are drugs or other substances that block the growth and spread of cancer by interfering with specific molecules or targets that are involved in the growth, spread, and progression of cancer. Targeted therapies are currently at the center of anti-cancer drug development; hence, they are the cornerstone of precision medicine. Similarly, in breast cancer, many drugs are being developed and integrated with nanocarriers. Table 3 lists some of the drugs used in breast cancer treatment along with nanocarriers responsive to ultrasound.

## 8. Design Considerations for Ultrasound Responsive Drug Carriers

Ultrasound-responsive nanocarrier design should consider a variety of design constraints, which can be broadly categorized as microbubble design considerations, ultrasound parameters, and cellular characteristics.

### 8.1. Microbubble

There are three main design considerations for achieving the preferred microbubble mechanism: bubble size and concentration, bubble-to-cell distance, and bubble shell material [106].

#### 8.1.1. Microbubble Size and Concentration

Ultrasound-responsive nanocarrier-based therapy has been shown to be successful in in vitro experiments [107]. However, under in vivo conditions, various physiological barriers should be overcome so that there is no hurdle for injected drugs in reaching their target sites [26]. In particular, therapeutic agents should first travel across blood vessels and then pass through the extravascular compartment prior to arriving at the target cells, which must then incorporate them [57]. To pass through these barriers, diffusion and convection are insufficient as the only driving forces, and sufficiently high driving forces are required. Cavitation plays an important role in this process [69]. The cavitation phenomenon in ultrasound-responsive nanocarrier-based therapy can not only create pathways through pore creation, but also enable therapeutic agents to travel within the target tissue with adequate momentum [43]. Two types of cavitation facilitate this process: stable and inertial [39]. In stable cavitation, the size of the gaps between endothelial cells is increased by mechanically pushing the walls of the blood vessels using microbubbles, thereby enabling drugs to enter the target tissue. Furthermore, stable cavitation enables the opening of particles, such as liposomal vesicles, which are employed in drug delivery [38]. Inertial cavitation, on the other hand, applies aggressive mechanical forces such as jetting and shockwaves that result from the disintegration of microbubbles. Thus, inertial cavitation enables the entrance of high levels of drugs into tumor tissues. It is a well-established fact that the cavitation characteristics of microbubbles are highly dependent on their size as higher dynamic responses can be stimulated around their resonant radius [108]. In conventional medical ultrasound cases with a frequency between 0.5 and 5 MHz, larger bubbles create stronger acoustic responses and increased membrane permeability than that by smaller bubbles [109]. However, it is sometimes challenging for microbubbles to pass through endothelial gaps in the range of 380–780 nm within tumor blood vessels [110]. In such cases, nanobubbles with a size range of 300–700 nm are used in place of microbubbles to achieve an enhanced permeability and retention (EPR) effect in tumors.

The number of bubbles around the target cell affects the extent of cavitation [111]. Experiments involving single cells undergoing cavitation have shown that the extent of cavitation tends to be less predictable if it is created by large bubbles (diameter greater than 5.5 µm) that exhibit translational movement over large distances. The number of bubbles around a cell is also positively proportional to the extent of cavitation, particularly for smaller bubbles (with diameters smaller than 5.5 µm) [112]. Furthermore, cavitation stimulated by the localized destruction of fewer than three bubbles is generally reversible, while those stimulated by the cavitation of four or more bubbles tend to be irreversible. In summary, experiments have shown that an increase in microbubble concentration decreases the microbubble cavitation threshold and improves treatment efficiency, with the tradeoff that cell viability would be compromised. Therefore, the concentration of microbubbles around the target cell must be carefully considered.

#### 8.1.2. Bubble-to-Cell Distance

The second important phenomenon that must be considered is microbubble dynamics with respect to different environments [113]. Subject to different environments, cavitation behaviors change substantially, which in turn drastically affects therapy efficacy [114]. The distance between two microbubbles and the distance between microbubbles and the boundary have a significant impact on cavitation [115]. Boundaries around microbubbles limit microbubble growth when confined, thus suppressing cavitation effects [17,115]. Microbubble cavitation triggered by ultrasound applies forces on tissue boundaries, thereby affecting microbubble behavior. Particularly, deformable environments closer to each other—such as micro-vessels—affect microbubble interactions, resulting in the reduction in microbubble expansion, restriction of fragmentation or jets during cavitation, and deformation of vessel walls, where the deformation is directly proportional to the microbubble size [115]. The interaction of microbubbles with each other following their administration into blood vessels can affect their cavitation, sonoporation, and overall therapeutic efficacy. Studies have reported that decreasing the inter-microbubble distance can also increase the microbubble lifetime [111]. Reducing the microbubble concentration also decreases the microbubble size and increases cavitation lifetime [111]. Furthermore, two or more microbubbles can be merged into a single microbubble by using high-pressure ultrasound energy [116]. The merging of microbubbles creates a large mechanical force that enables the creation of larger pores, but there is a possibility that it may result in tissue damage. However, microbubble fusion without the presence of ultrasound suffers from the issue of reduced microbubble circulation time. This challenge has been resolved using a phenomenon called PEGylation, whereby polyethylene glycol groups are added to prevent aggregation [117].

#### 8.1.3. Bubble Shell Material

In ultrasound-responsive nanocarrier-based therapy, the microbubble shell material is an important design constraint to consider [118]. Few studies have compared the therapeutic efficiency of various microbubbles covered with different types of shells [119]. Generally, lipid-shelled microbubbles yield a higher therapeutic ratio than that by protein-shelled microbubbles. A few studies have been carried out to design improved microbubble shells, thereby enhancing therapeutic efficacy [120]. These include biodegradable microcapsules and integrating nanoparticles with microbubbles [121]. Furthermore, studies involving the properties of microbubbles—such as stiffness, scattering, and thermal effects— have been conducted with respect to the application of different shell materials. Peng et al. [122] showed that it is possible to produce caged microbubbles with varying shell elasticity and porosity so that it will be possible to adjust resonance frequency and regulate cavitation modes.

### 8.2. Ultrasound Parameters

Ultrasound-responsive nanocarrier-based therapy design should also consider ultrasound parameters—such as frequency, intensity, mechanical index (MI), and exposure time—as they are high determinants of treatment efficacy [46].

#### 8.2.1. Frequency

The cavitation behavior of microbubbles at a given frequency is strongly dependent on their size because the response of the microbubbles is much larger around their resonance radius [36]. As such, a drive frequency of 1 MHz was used, as this frequency is in principle closer to the resonant frequency of commercially available microbubbles, which typically range in diameter from 1 to 3 µm [55]. However, for microbubble solutions with a relatively wide size distribution, the sonoporation efficiency increases at lower drive frequencies. Generally, for therapeutic applications, the frequency of ultrasound is lower than that used for diagnostic purposes. A lower ultrasonic frequency ensures deeper tissue penetration owing to reduced attenuation, leading to optimal therapeutic outcomes [123]. The ultrasonic frequency used also depends on the type of microbubbles used as the use of an ultrasonic frequency near or similar to the resonant frequency of the microbubbles promotes stable microbubble cavitation [124]. However, in the case of higher sound pressures causing inertial cavitation, the frequency used becomes a less important consideration, as microbubbles collapse under high ultrasound pressure.

#### 8.2.2. Intensity

As high-intensity ultrasound can potentially cause tissue alterations due to ultrasound-associated heating effects, the Food and Drug Administration (FDA) has set the intensity at a level that causes less than a 1 °C rise in temperature [125]. Typically, the ultrasonic intensity range for delivery applications is 0.3–3 W/cm^2^. However, higher-intensity ultrasound can be used when the pulse length (pulse period/ultrasound frequency) and/or pulse repetition frequency (pulses/s) are reduced, resulting in a low duty cycle (pulse length × pulse repetition frequency); therefore, the time-averaged intensity (duty cycle × ultrasonic intensity) decreases [46,55].

#### 8.2.3. Mechanical Index (MI)

The MI of ultrasound is a measure of the peak negative pressure (MPa) per square root of the center frequency (MHz). MI is a common alternative to ultrasound intensity because it measures the acoustic pressure applied to the tissue [32]. The MI provides a direct measure of the amount of cavitation that occurs. Higher MI values result in higher cavitation activity [48]. To avoid any unwanted thermal effects during therapy, MI usually ranges from 0.2 to 1. The FDA has set the maximum intensity level (MI) for clinical ultrasound applications at 1.9 to minimize direct tissue damage [126].

#### 8.2.4. Exposure Duration

The exposure duration is another important factor that may affect the extent of sonoporation because the accumulated acoustic energy delivered to cells is equal to the product of the acoustic intensity and the total exposure duration. Shorter pulse lengths are often suggested as a way to reduce the cavitation-induced shear stress responsible for membrane pore generation [127]. In the case of single-pulse ultrasound exposure, the pulse length is the same as the exposure time, which has been shown to be proportional to treatment efficiency and negatively proportional to cell viability [128]. Cavitation can also be effectively achieved with very short pulses if high ultrasound pressure is applied [129]. The duration of therapy for ultrasound-responsive nanocarrier-based treatment should be guided by the time required for ultrasound to induce inertial or stable cavitation and sonoporation while avoiding unwanted thermal effects [130]. In addition, the duration of the ultrasound application depends on the type and location of the tissue to be treated, and the type of microbubble used in the applied ultrasound intensity. Under high pressure, immediate inertial cavitation, multiple or continuous injections of microbubbles, and prolonged treatment time can improve the efficiency [131]. Similarly, the time needed for the optimized movement of microbubbles also needs to be considered because prolonged treatment times at low pressures might also result in heating effects [132].

### 8.3. Cellular Characteristics

Nanocarrier-based therapy occurs in living cells, and it is clear that therapy efficiency is dependent on cellular properties. Cellular properties—such as cell density, cell types, and different cell cycle phases—affect different phenomena that occur in nanocarrier-based therapy, including cavitation and sonoporation.

#### 8.3.1. Cell Type Variations

Different cell types are known to exhibit different responses to cavitation; varying levels of treatment efficacy and cell viability responses have been recorded while using different cancer cell lines in experiments involving the efficacy of ultrasound-responsive nanocarrier-based treatment [133]. For example, variations in bioeffects have been observed across different cancer cell types, including breast, liver, ovarian, and thyroid cancer cells. Cell types have also been shown to affect the duration of cavitation, as varying times have been recorded for different cell types [134]. Therefore, cell type variation should be considered when designing nanocarriers for breast cancer therapy.

#### 8.3.2. Cell Cycle Dependence

Cells that undergo cavitation in the G2 (growth and preparation for mitosis) and M (mitosis) phases have been observed to have higher treatment efficiency than cells in the G0 (resting), G1 (growth), or S (DNA synthesis) phases [135,136]. Another study showed that S-phase cells that undergo deoxyribonucleic acid (DNA) synthesis exhibit higher levels of drug uptake in direct response to cavitation [137]. This warrants a thorough examination of the cell cycle when designing ultrasound-responsive nanocarrier-based treatments.

#### 8.3.3. Biochemical Effects

The extracellular fluid affects how a cell responds to a sonoporation episode. For example, extracellular calcium (Ca^2+^) is known to be important in the repair of cavitation sites, since the absence of this ion in the extracellular space critically prevents the cavitation site from initiating repair [48]. In contrast, the addition of synthetic nanoparticles to the extracellular space can modulate the efficiency of gene transfection [130]. Specifically, it has been shown that the addition of polyethylene amine (PEI) to the extracellular fluid can effectively prolong gene expression, thereby enhancing treatment efficiency. In studies involving cell culture, cavitation may be affected by the culture environment [138]. To better reproduce the biological effects of cavitation activity, studies were performed within the entire volume of medium mixed with cells in suspension. However, stronger targeted microbubble attachment and more vigorous bubble oscillation were observed for cells cultured on rigid substrates, and higher pressures may be required to generate cavitation in the cell membranes cultured on soft substrates. Attempts have also been made to study the results of cavitation using monolayer cell samples to simulate the in vivo tissue environment.

## 9. Challenges and Future Directions

### 9.1. Challenges

Combining ultrasound with nanoparticles has been shown to increase the treatment efficacy in breast cancer chemotherapy. Ultrasound-mediated therapeutic agent delivery leverages the enhanced permeability of cell membrane through the sonoporation. Increased permeability allows more effective delivery of therapeutic agents through solid tumors and other physiological barriers. There are a few FDA approved treatment methods utilizing ultrasound responsive nanocarriers for breast cancer. Moreover, there are many clinical trials being carried out that apply ultrasound responsive nanocarriers for breast cancer treatment. Nevertheless, there are concerns regarding the utilization of ultrasound based nanocarriers for breast cancer treatment. Ultrasound energies surpassing certain amount can disrupt the cell membrane. Uncontrolled usage of ultrasound may result in damage to nanocarriers and the therapeutic agent as well as other side effects to the normal cells. Furthermore, ultrasound is not effective for use in all environments, therefore it needs optimization depending on the presence of gas within tissues acting as obstacle for propagation of ultrasound waves.

Breast tumors possess biological characteristics which are divergent from normal cells/tissues. The tumors and tumor micro-environment may possess physically compromised vasculature, unusual extracellular matrix, and high interstitial fluid pressure that are all challenges that must be overcame by nanocarriers for therapy efficacy. Nanocarriers must penetrate the extracellular matrix of the tumors given the challenges of abnormal blood flow, impaired venous and lymphatic drainage, and irregularity of vasculature all affecting the effective diffusion of therapeutic agents to target tumor. Liposomes are the widely used nanocarriers in breast chemotherapy due to their biodegradability, biocompatibility, and their physical properties. Even though liposomes suppress internal toxicity, they could not improve efficacy. Liposomes are still struggling to balance between drug bioavailability and prolonged stability. Dendrimers on the other hand allow for more flexible design and adaptation, but dendrimers have a drawback of cytotoxicity. Micelles pose challenges with toxicity, instability, and chronic inflammation, but preferred for specific targeted therapy. The more relevant challenge involving micelles in ultrasound responsive nanocarrier application is poor tissue penetration capability.

### 9.2. Future Directions

In ultrasound responsive nanocarrier based therapy, it is important to consider safety. Ultrasound parameters should be optimized. Currently, there are some thresholds used based on FDA standard, but more studies considering ultrasound parameters are needed to be set, which are application or disease specific. Furthermore, nanocarrier design should consider more degradable materials while increasing efficacy. For instance, improvements in mapping out, synthesizing, and amending biodegradable polymers should be the work to focus on in the future to improve therapy efficacy and minimize adverse effects. Regarding dendrimers, the work to follow will be to amend the shell with low toxicity materials to adjust them to physiological factors.

Generally, future studies should focus on the way to provide a safer therapeutic method while having a localized control of the drug effect. Moreover, current studies are limited to in-vitro and animal model-based in-vivo studies. These studies should be translated to clinical studies.

## 10. Other Breast Cancer Treatment Applications of Nanocarriers

Nanocarriers are not only used in breast chemotherapy, but also in radiotherapy, photothermal and photodynamic therapy, and surgical interventions (cryosurgery) [139,140].

**Figure 8:**
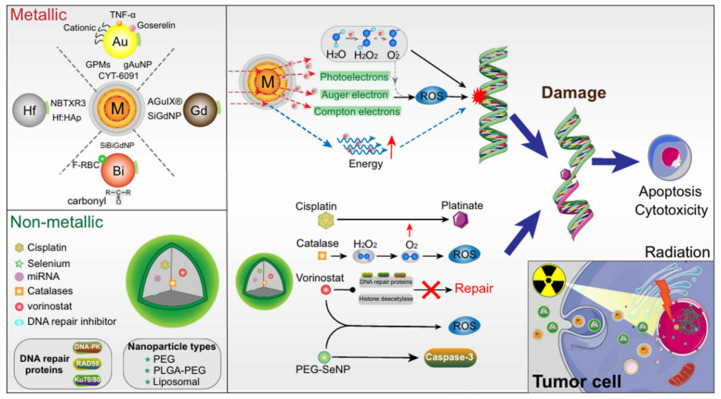
Mechanism of nanocarrier based radiotherapy. Adapted from Jin et al. [145] with permission under the terms of the CC BY 4.0 License, Copyright 2020.

Radiotherapy utilizes ionizing radiation produced by rays to treat tumor by killing local cells [141]. It involves the precise application of high intensity ionizing radiations to the tumor tissue leading to the death of tumor cells, as shown in Figure 8. Radiotherapy is primarily used to treat primary and metastatic solid tumors [142]. However, radiation therapy has limitations [143,144]. First, there is the possibility of harm to the surrounding healthy tissue [139]. Second, tumor cells at distant from the radiation site are subjected to low intensity radiation, which affects the efficacy of treatment [143]. Thirdly, tumor cells sometimes develop resistance to the ionizing radiation due to which increased dosage is utilized resulting in the death of surrounding normal tissue [139,140,143,144]. Though an important milestone has been achieved in better focusing and more regulated dosage of ionizing radiation, radiation resistance and inherent flaws of therapeutics remain a challenge [139]. Studies suggested different approaches, including enhancing radio sensitization of tumor tissue, enhancing radio resistance of healthy tissue, and reversal of radiation resistance in tumor tissue; all of which were made possible via nanocarriers [144,145]. Nanoparticle materials used in radiotherapy include precious metals, iron oxides, and semiconductors [146]. Precious metals nanocarriers are high atomic number metals, including gold, silver, gadolinium, hafnium, platinum, and bismuth. Gold nanocarriers are popular due to their chemical stability, biocompatibility, and strong photoelectric absorption coefficient. Iron oxide nanocarriers have been shown to improve image-guided radiotherapy by enhancing radiotherapy dose [147]. Likewise, semiconductor nanoparticles—especially mesoporous silica—have been shown to enhance radiotherapy by producing fine hydroxyl radicals that kill tumor cells effectively [148,149].

Phototherapy is a type of medical treatment where light is utilized to treat conditions including cancer and peripheral infections [150]. The two types of phototherapy now employed for the treatment of diseases are photothermal therapy (PTT) and photodynamic therapy (PDT) [151]. In PDT, the treatment is carried out through a sequence of photochemical reactions triggered by photoactivated molecules or materials known as photosensitizer (PS) medications. In PTT, a photothermal (PT) agent is used for the selective local heating for repairing aberrant cells or tissues. Currently, PDT and PTT based on nanoparticles (NPs) have demonstrated significant efficacy, modest invasion, and few side effects during tumor treatment [139,140,150]. Using a novel class of light-to-heat conversion nanomaterials, cancer cells can be killed by converting light energy into heat energy. This has many advantages compared to the conventional photothermal conversion materials [139]. The first advantage is that—through particle surface modification—nanoparticles can produce the impact of tumor-targeted aggregation, which increases the target tumor’s capacity for enrichment. Secondly, the use of nanoparticles enables better imaging capability than conventional photothermal materials. Thirdly, reversion of multidrug resistance can be achieved by nanoparticle mediated phototherapy. Additionally, by compromising the integrity of tumor cell membranes, nanoparticle-mediated PTT can improve the efficacy of therapy.

**Figure 9:**
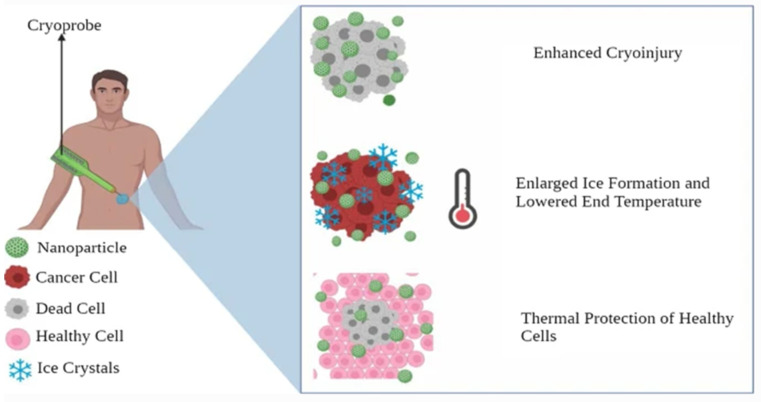
Nanocarriers in cryosurgery. Adapted from Gavas et al. [139] with permission under the terms of the CC BY 4.0 License, Copyright 2021.

In cryosurgery procedure, abnormal tissue is frozen and destroyed by using an instrument called cryoprobe or an extremely cold liquid [152], as seen in Figure 9. Cryosurgery has advantages, such as low invasiveness, less bleeding, less postoperative complications, and low cost [153]. However, cryosurgery has limitations, including inadequate freezing effectiveness and freezing injury to neighboring tissues [139]. As nanotechnology advanced, the idea of nano-cryosurgery was put out. Nanoparticles (NPs) having particular physical or chemical properties are introduced into tumor tissues as the core working principle of nano-cryosurgery [154]. Not only can the efficiency and efficacy of freezing be increased by making use of nanocarriers’ unique qualities, but also the direction of ice ball production and range adjustment. Breast tumor cryotherapy may be a practical way to get rid of malignant breast tumors [152]. Due to the superficial localization of the glands, which makes them accessible for both cryo-probes and imaging tools such as ultrasound (US), as well as the lack of intervening vital organs that could be damaged during the procedure and cause serious complications, the removal of breast tumors through cryoablations appears to be feasible [152,154,155].

## Figures and Tables

**Table 1:** Advantages and disadvantages of internal and external stimuli.

Stimuli	Advantages	Disadvantages
Internal	Safe and efficient	Cannot be manually controlled
	Controllable release	
	Protect cells and hinders cellular apoptosis	
	Efficient drug release without compromising specificity	
External	Can be manually controlled and modulated based on individual requirements	Several types of specialized equipment and techniques needed
	Provide upgraded site-specific drug delivery	Normal cell injury
	Constant and rapid payload release	

**Table 2:** Studies related to ultrasound responsive nanocarriers for breast cancer treatment.

Nanocarrier	Platform	Outcome	Drug	Reference
Micelles	Pluronic P123/F127	Longer circulating time and increased cellular uptake	Curcumin	[29]
Polymeric micelle	Polyethylene glycol (PEG)-polylactic acid (PLA)	Increased cell death, minimal effect on normal cells, greater surface area, and larger number of particles generated by ultrasound	Doxorubicin	[57]
Oxygen	SE61 microbubbles recharged with oxygen or nitrogen	Increase breast tumor oxygenation levels, enabling oxygen delivery to avascular regions of the tumor, improvements in tumor growth and animal survival	Radiation	[58]
Liposome	SonoVue and saline	Ultrasound enabled to see accurately how a tumor responds to therapy	Docetaxel, epirubicin, cyclophosphamide	[59,60,61,63]
Carbon	Perfluorocarbon nanoemulsions	Excellent anti-cancer effects characterized by tumor regression, and displayed long-lasting, strong ultrasound contrast	Doxorubicin	[37]
Liposome	Sonazoid and perfluoromethylcylopentan	Acoustic cluster therapy induces a strong increase in the therapeutic efficacy	Doxorubicin	[64]
Oxygen	Span 60 and water-soluble vitamin E	Rapid tumor growth after treatment, increase in volume, delay tumor progression, and improve survival in a murine model of metastatic breast cancer	Radiation	[65]
Magnetic	Polylactic-co-glycolic acid (PLGA)	Excellent imaging performance and good biocompatibility	-	[66]
Liposome	Perfluoropentan	Adequate drug release, deep tumor penetration, well-controlled release of drug, and elevated antitumor efficacy	Doxorubicin	[67]

**Table 3:** Drugs used in ultrasound-responsive nanocarrier breast cancer treatment.

Drug	Product/Platform	Type of Nanocarrier	Reference
Doxorubicin	Perfluoropropane	Liposome	[69,70]
Doxorubicin	Polyethylene glycol	Liposome	[71]
Doxorubicin	Polyethylene glycol	Liposome	[72]
Cisplatin	Soy phosphatidyl choline (SPC-3), cholesterol, dipalmitoyl phos-phatidyl glycerol (DPPG), and methoxy-polyethylene glycol-distearoyl phosphatidylethanolamine (mPEG 2000-DSPE)	Liposome	[73]
EndoTAG-1 and paclitaxel	Cationic	Liposome	[74]
Paclitaxel	1,2-dioleoyl-sn-glycero-3-phosphocholine	Liposome	[75]
Resveratrol	Chloroform solutions of cadmium oxide and sucrose laurate	Liposome	[76]
Cisplatin	Distearoyl phosphoethanolamine-polyethylene glycol and phosphatidylcholine	Liposome	[77]
Paclitaxel	Polyethyleneglycol (PEG)-phosphatidylethanolamine (PE) (PEG-PE)	Liposome	[78]
Doxorubicin and silymarin	3-(4,5-dimethylthiazol-2-yl)-2,5-diphenyltetrazolium bromide (MTT)	Liposome	[79]
Epirubicin-hydrochloride	Phosphatidylcholines with thin film hydration using egg yolk	Liposome	[80]
Curcumin	Polyethylene glycol (PEG)	Liposome	[81]
A7R-cysteine peptide	Distearoylphosphosphatidyl-ethanolamine (DSPE-PEG2000)	Liposome	[82]
Raloxifene	Methanol-ethyl acetate	Liposome	[83]
Artemisinin	Polyethylene glycol 2000 (PEG 2000)	Liposome	[84]
Thymoquinone	Thymoquinone (2-isopropyl-5-methyl-1,4-benzoquinone) and Triton X-100; 1,2-dipalmitoyl-sn-glycero-3-phospho-choline (DPPC)	Liposome	[85]
Doxorubicin	Lipoic acid, hyaluronic acid, L-lysine methyl ester	Polymer nanoparticles	[86]
Doxorubicin	Chitosan and pluronic F127	Polymer nanoparticles	[87]
Cisplatin	Luteinizing hormone-releasing hormone (LHRH)-modified dextran	Polymer nanoparticles	[88]
Tamoxifen citrate	Polylactide-co-glycolide	Polymer nanoparticles	[89]
Paclitaxel	Albumin nanoparticle	Polymer nanoparticles	[90]
Paclitaxel	Folic acid Polylactic-co-glycolic acid, polyethylene glycol succinate	Polymer nanoparticles	[91]
Paclitaxel	Montmorillonite and Poly(D, L-lactide-co-glycolide)	Polymer nanoparticles	[92]
Paclitaxel and ceramide	Poly(beta-amino ester) and poly(D,L-lactide-co-glycolide)	Polymer nanoparticles	[93]
Docetaxel	Albumin nanoparticle	Polymer nanoparticles	[94]
Quercetin	Polylactic-co-glycolic acid, polyethylene glycol 1000 succinate	Polymer nanoparticles	[95]
Doxorubicin and Salinomycin	Polyacrylic acid and Polyethylene glycol	Micellar nanoparticle	[96]
Paclitaxel	Polyethylene glycol succinimidyl succinate	Micellar nanoparticle	[25]
Doxorubicin and Paclitaxel	Lauryl carbamate derivative of plant-based polymer inulin	Micellar nanoparticle	[97]
Paclitaxel	Polyethylene glycol-b-polylactide	Micellar nanoparticle	[24]
Fisetin	Pluronic127 folic acid	Micellar nanoparticle	[98]
Paclitaxel	Dextran-g-indomethacin	Micellar nanoparticle	[99]
Aminoflavone	Anti-epidermal growth factor receptor	Micellar nanoparticle	[100]
Paclitaxel	PEG-block-poly[(1,4-butanediol)-diacrylate-β-5-amino-1-pentanol] polyethyleneimine-block-PDHA	Micellar nanoparticle	[101]
Aminoflavone	Poly(amidoamine) dendrimer, polyethylene glycol derivatives	Micellar nanoparticle	[102]
Doxorubicin	Pluronic copolymer P123 polyethylene glycol-block-poly (di-isopropanolamino ethyl methacrylate) diblock copolymer	Micellar nanoparticle	[103]
Paclitaxel	Methoxy polyethylene glycol-polylactide (mPEG-PLA)	Micellar nanoparticle	[104]
Paclitaxel	polyethylene glycol (PEG)-polyacrylic acid (PAA) (PEG-PAA)	Micellar nanoparticle	[105]

## Data Availability

Not applicable.
